# Dietary Choline Intake Is Beneficial for Cognitive Function and Delays Cognitive Decline: A 22-Year Large-Scale Prospective Cohort Study from China Health and Nutrition Survey

**DOI:** 10.3390/nu16172845

**Published:** 2024-08-26

**Authors:** Feifei Huang, Fangxu Guan, Xiaofang Jia, Jiguo Zhang, Chang Su, Wenwen Du, Yifei Ouyang, Li Li, Jing Bai, Xiaofan Zhang, Yanli Wei, Bing Zhang, Yuna He, Huijun Wang

**Affiliations:** 1 National Institute for Nutrition and Health, Chinese Center for Disease Control and Prevention, Beijing 100050, China; huangff@ninh.chinacdc.cn (F.H.); guanfx@ninh.chinacdc.cn (F.G.); jiaxf@ninh.chinacdc.cn (X.J.); zhangjg@ninh.chinacdc.cn (J.Z.); suchang@ninh.chinacdc.cn (C.S.); duww@ninh.chinacdc.cn (W.D.); ouyyf@ninh.chinacdc.cn (Y.O.); lili@ninh.chinacdc.cn (L.L.); baijing@ninh.chinacdc.cn (J.B.); zhangxf@ninh.chinacdc.cn (X.Z.); weiyl@ninh.chinacdc.cn (Y.W.); zhangbing@chinacdc.cn (B.Z.); heyn@ninh.chinacdc.cn (Y.H.); 2 NHC Key Laboratory of Public Nutrition and Health, Beijing 100050, China

**Keywords:** dietary choline, cognition, cognitive decline, elderly, cohort study, CHNS

## Abstract

Pre-clinical studies have discovered the neuroprotective function and the benefit for cognitive function of choline. However, it remains unclear whether these benefits observed in animal studies also work in humans. The aims of this study are to examine the effects of dietary choline intake on cognitive function and cognitive decline during ageing in middle-aged and elderly Chinese. We included 1887 subjects aged 55~79 years with 6696 observations from the China Health and Nutrition Survey cohort study. The subjects were followed up for 6 to 21 years, with an average of 12.2 years. A dietary survey was conducted over 3 consecutive days with a 24 h recall, using household weight-recording methods. Based on the China Food Composition, data from USDA, and published literature, the dietary choline intake was calculated as the sum of free choline, phosphocholine, phosphatidylcholine, sphingomyelin, and glycerophosphocholine. Cognitive function was assessed using a subset of the Telephone Interview for Cognitive Status-modified (TICS-m) items. In order to eliminate the different weight of scores in each domain, the scores were converted by dividing by the maximum score in each domain, which ranged from 0 to 3 points. Higher cognitive scores represented better cognition. We used two-level mixed effect models to estimate the effects of dietary choline intake on cognitive score and cognitive decline rate in males and females, respectively. The average dietary choline intake was 161.1 mg/d for the baseline. After adjusting for confounders, the dietary choline intake was significantly associated with higher cognitive score in both males and females. The cognitive score in the highest quartile group of dietary choline was 0.085 for males and 0.077 for females–higher than those in the lowest quartile group (*p* < 0.01 for males, *p* < 0.05 for females). For every 10-year increase in age, the cognitive score decreased by 0.266 for males and 0.283 for females. The cognitive score decline rate of the third quartile group of dietary choline was 0.125/10 years lower than that of the lowest quartile group in females (*p* < 0.05). Dietary choline intake not only improves cognitive function, but also postpones cognitive decline during the aging process. The findings of this study highlight the neuroprotective benefit of choline in the middle-aged and elderly Chinese population, especially among females.

## 1. Introduction

In China, it has been reported that the prevalence of dementia increases with age, from 2.9% in individuals aged 60–69 years to 31.9% in those aged 90 years or older, and the prevalence of MCI increases from 11.9% in people aged 60–69 years, to 33.1% in those aged 90 years or older [1]. The seventh national population census conducted in 2020 showed that the number of people aged 60 and above in China has exceeded 260 million, accounting for 18.70% of the total population, of which 13.50% are aged ≥65. China has currently entered a rapidly aging society, suggesting a heavy burden of cognitive decline on the individual, family, and society.

Choline is a vitamin-like essential macronutrient for human beings [2,3,4,5,6,7]. Although humans can produce a small amount of choline through the hepatic phosphatidylethanolamine N-methyltransferase pathway, most people still need to increase their dietary choline intake to prevent deficiency [4]. In foods, it is found in both water-soluble (free choline, phosphocholine, and glycerophosphocholine) and lipid-soluble (phosphatidylcholine and sphingomyelin) forms [4]. For all healthy adults, the European Food Safety Authority (EFSA) set an AI of 400 mg/d in 2016 [4]. Chinese DRIs (2013) originally set an AI of 500 mg/d and 400 mg/d for men and non-pregnant women in adult Chinese [8]. Subsequently, Chinese DRIs (2023) decreased the AI to 450 mg/d for men and 380 mg/d for non-pregnant women [9]. Choline is a precursor of different metabolites, such as neurotransmitter acetylcholine (ACh), the membrane phospholipid phosphatidylcholine, and sphingomyelin, as well as the methyl donor betaine [2,9]. Therefore, it is implicated in the proper functioning of liver, muscle, brain, and kidneys throughout the lifespan [2].

Previous pre-clinical studies have found the neuroprotective function and the benefit for cognitive function of choline [2,3,6,7,10]. However, it remains unclear whether these benefits observed in animal studies also work in humans. The epidemiological studies on choline intake and cognitive function are limited, and the results are mixed. In an observational study of 2796 adults aged 60 years and older from the National Health and Nutrition Examination Survey (NHANES) 2011–2012 and 2013–2014 waves, neither dietary choline intake (OR = 0.94, 95% CI = 0.75~1.17) nor total choline intake (OR = 0.87, 95% CI = 0.70~1.09) was associated with cognitive scores [11]. However, another study on 2393 adults aged 60 years and older, who were also from the NHANES project (the 2011–2012 and 2013–2014 waves, as before) showed that, compared to the lowest tertile of total choline intake (<187.60 mg/d), only the medium tertile of total choline intake (187.60–399.50 mg/d) illustrated a protective effect on cognitive function, and there was no statistical significance in the highest tertile of total choline intake [12]. A prospective, population-based Kuopio Ischemic Heart Disease Risk Factor Study (KIHD) involving 2497 dementia-free men aged 42~60 years old in 1984~2989 in eastern Finland showed that the total choline intake was not associated with the risk of incidence of dementia, the risk of Alzheimer’s disease (AD), or cognitive performance, and higher phosphatidylcholine intake was associated with a lower risk of incidence of dementia and better cognitive performance [13]. Another study of 1391 adults aged 36~83 years from the Framingham Offspring cohort found that the dietary choline intake was positively associated with verbal memory (β = 0.60, 95% CI = 0.29~0.91) and visual memory (β = 0.66, 95% CI = 0.19~1.13), and was not associated with verbal learning (β = 0.14, 95% CI = −0.23~0.51) or executive function (β = 0.14, 95% CI = −0.11~0.39) [14].

In conclusion, there has been a great dearth of epidemiological studies on choline intake and cognition function in community-based cohorts. Given the lack of evidence from the Chinese population, the aim of this study is to evaluate the effect of dietary choline intake on the cognitive function and cognitive decline rate in middle-aged and elderly Chinese adults.

## 2. Materials and Methods

### 2.1. Study Design and Population

The data used in this study were from the China Health and Nutrition Survey (CHNS). This project was carried out in collaboration with National Institute for Nutrition and Health of the Chinese Center for Disease Control and Prevention and the University of North Carolina at Chapel Hill in the United States. It was a long-term, large-scale open cohort study that covered a total of sixteen provinces (autonomous regions and municipalities). The first round of investigation began in 1989, and was followed-up in 1991, 1993, 1997, 2000, 2004, 2006, 2009, 2011, 2015, and 2018 respectively. The more detailed information about CHNS was described previously [15,16].

Given that the cognitive function was evaluated in the 1997, 2000, 2004, 2006, 2015, and 2018 waves, participants aged 55~79 years old in the aforementioned years were included in this study, and those with incomplete dietary data, cognitive function score, unreasonable energy intake (>5000 kcal/d or <800 kcal/d for males, >4000 kcal/d or <600 kcal/d for females), and unreasonable dietary total choline intake (<1st percentile or >99th percentile) were excluded. For the current analysis, the baseline was the year in which the participants were first investigated.

The institutional review board of the National Institute for Nutrition and Health of Chinese Center for Disease Control and Prevention approved the study protocol (ethics approval code 2018-004, 14 March 2018). All the participants signed the informed consent forms.

### 2.2. Methods of Investigation

#### 2.2.1. Assessment of Dietary Choline Intake

In each wave of the survey, a dietary recall method over 3 consecutive days and a 24 h-period (including 2 workdays and 1 weekday) was employed to collect individual consumption of all food and drink. A household weighing method was used to collect the household consumption of cooking oil and condiments over the corresponding 3 days.

Due to the fact that only some foods in the China Food Composition have choline content, we compiled and constructed a complete choline content database based on USDA [17], and the published literature, including free choline, phosphocholine, phosphatidylcholine, sphingomyelin, glycerophosphocholine, and betaine. The total choline content is the sum of free choline, phosphocholine, phosphatidylcholine, sphingomyelin, and glycerophosphocholine.

According to the quartiles, the total dietary choline intake was categorized into the lowest-intake group (<25th percentile, Q1), lower-intake group (25–50th percentile, Q2), higher-intake group (50–75th percentile, Q3), and highest-intake group (>75th percentile, Q4).

#### 2.2.2. Cognitive Function

The cognitive function was assessed using a subset of the Telephone Interview for Cognitive Status-modified (TICS-m) items by face-to-face interview [18]. TICS-m is a well-established and widely used screening instrument for dementia and assessment of global cognitive function in older people [19,20,21,22,23,24]. Although it is designed for telephone interview, the survey involving memory, attention, and calculation domains was completed by face-to-face interview. Memory was assessed by immediate and delayed recall of a list of 10 words (10 points each). Attention was evaluated by counting backward from 20 to 1 (2 points). Calculation was estimated by serial 7 subtraction (5 points). The original total cognitive score was calculated as the sum of the 3 domains, and ranged from 0 to 27. In order to eliminate the different weight of scores in each domain, the scores were converted by dividing by the maximum score in each domain, and they ranged from 0 to 3 points. Higher cognitive scores represented better cognition.

#### 2.2.3. Covariates

The education level was divided into three groups, namely primary school and below, junior high school, and high school and above. Smoking was grouped into non-smoking (never smoked and currently quitting smoking) and current smoking. Income refers to the annual household income per capita, which was divided into three tertiles: low-income group, middle-income group, and high-income group. The criteria for determining hypertension were: (1) diagnosed by the doctor, or (2) currently taking anti-hypertensive medicine, or (3) the average high blood pressure measured by physical examination of ≥140 mmHg or the average low blood pressure of ≥90 mmHg. The criteria for DM were: (1) diagnosed by the doctor, or (2) taking hypoglycemic medicine, or (3) injecting insulin, or (4) according to the standards of the Chinese Guidelines for the Prevention and Treatment of Type 2 diabetes (2020 Edition), venous fasting blood glucose ≥ 7.0 mmol/L or venous fasting glycosylated hemoglobin ≥ 6.5%.

### 2.3. Statistical Analysis

The continuous variables with normal distribution are described as mean ± standard deviation, while continuous variables with non-normal distribution are described as median (P_25_, P_75_). Categorical variables are presented as proportion. Given the data hierarchy caused by multiple measurements, two-level mixed effect models were used to estimate the effects of dietary choline on cognitive function. Age was re-scaled by dividing by 10 to facilitate the interpretation of the regression coefficients.

Confounders in the analyses were selected based on previous studies. Model 1 was adjusted for age and education. Model 2 controlled variables in model 1, namely income, urban/rural, energy intake, physical activity, hypertension, DM, stroke, smoking, and alcohol intake. The interaction term between age and dietary choline intake was included in each model to evaluate the effects of dietary choline intake on the cognitive decline over time. Tests of linear trend of cognitive function over time were conducted by restricted cubic spline. All analyses were stratified by sex. All *p*-values were 2-tailed (α = 0.05). Data cleaning was completed by SAS 9.4, and data analysis and plotting were performed using R 4.4.0.

## 3. Results

### 3.1. Characteristics of the Study Population

A total of 1887 subjects aged 55~79 years with 6696 observations were analyzed in this current study. There were 1068, 638, 146, and 35 subjects who participated in 3, 4, 5, and 6 waves of investigation, respectively. The subjects were followed up for 6 to 21 years, with an average of 12.2 years. As shown in Table 1, the mean age was 60.1 ± 4.4 years for the baseline of the cohort, and 50.7% were males. The proportion of smoking and drinking had been decreasing, with smoking decreasing from 34.4% in 1997 to 18.9% in 2018, and drinking decreasing from 34.8% in 1997 to 19.1% in 2018. The household annual income per capita was on the rise year by year. For the baseline of the cohort, the median intake of dietary choline was 161.1 mg/d.

### 3.2. Effects of Dietary Choline Intake on Cognitive Function

The dietary choline intake was evenly divided into four groups from low to high, namely lowest, lower, higher and highest, with an average intake of 60.4, 125.6, 192.6, and 312.8 mg/d for males, and 59.0, 121.5, 189.9, and 298.5 mg/d for females.

**Figure 1:**
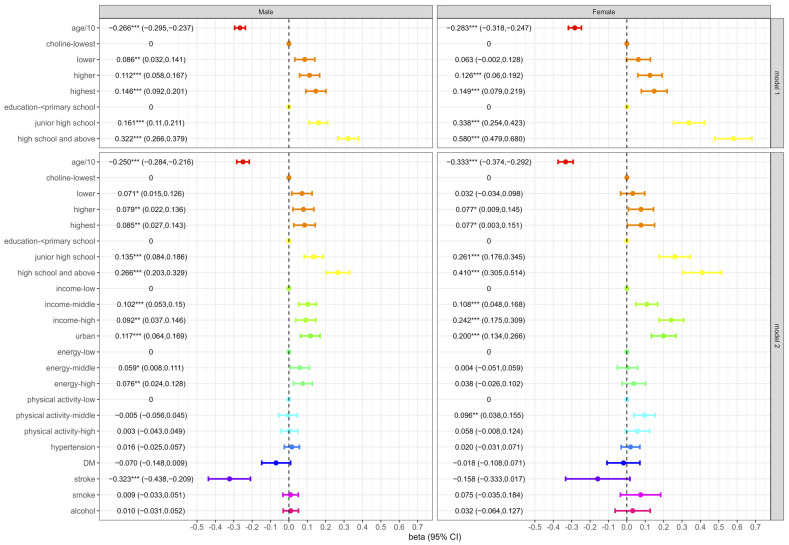
The effects of dietary choline intake on cognitive function score. *: *p* < 0.05; **: *p* < 0.01; ***: *p* < 0.001.

Figure 1 showed the effects of dietary choline intake on cognitive function. Model 1 was adjusted for age and education, and model 2 was additionally adjusted for income, urban/rural, energy intake, physical activity, hypertension, DM, stroke, smoking, and alcohol intake based on model 1. Model 1 showed that the cognitive scores in the highest intake group for males and females were 0.146 (*p* < 0.001) and 0.149 (*p* < 0.001) higher than those in the lowest intake group, respectively. *p*-values of the trend test for males and females were both <0.0001. After being adjusted for covariates, the results were still significant.

As shown in model 2, the cognitive scores in the highest intake group for males and females were 0.085 (2.83% of the total score) and 0.077 (2.57% of the total score) higher than those in the lowest intake group, respectively. *p*-values of the trend test for males and females were 0.0067 and 0.0188, respectively. For every increase of 10 years in age, cognitive scores decreased by 0.250 and 0.333 points for males and females, respectively (both *p*-values < 0.001).

### 3.3. Effects of Dietary Choline Intake on Cognitive Decline Rate

As age increased, cognitive scores showed a significant linear decline tested by the restricted cubic splines for different sexes. Both of the *p*-values of overall association for males and females were <0.0001, while the *p*-values of non-linear association were 0.1531 and 0.4665, respectively.

**Figure 2:**
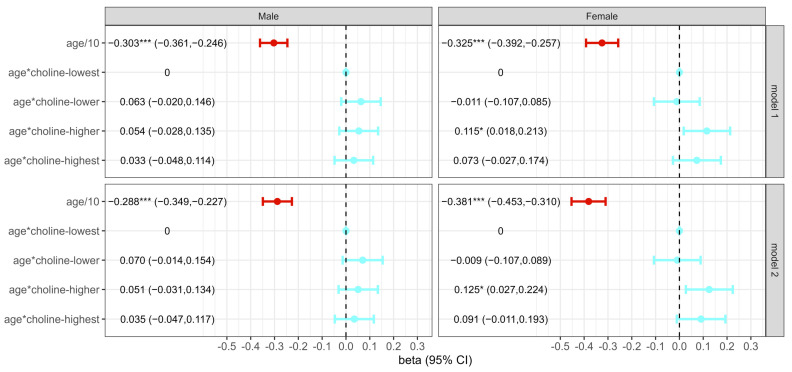
The effects of dietary choline intake on cognitive decline. *: *p* < 0.05; ***: *p* < 0.001.

Figure 2 showed the effects of dietary choline intake on cognitive decline rate. In model 1, after being adjusted for education, the interactive item between age and dietary choline intake was not statistically significant in males. While in females, the decline rate of cognitive score every 10 years in the higher intake group was 0.115 (3.83% of the total score) higher compared to the lowest intake group (*p* < 0.05).

After controlling for other covariates, the interactive items were still not statistically significant in males, and the coefficients were 0.070, 0.051, and 0.035 in the lower, higher, and highest intake groups, respectively (all *p*-values > 0.05). The *p*-value of the trend test was 0.5127. In females, the cognitive score decline rate of the higher intake group of dietary choline was 0.125 points/10 years lower than that of the lowest intake group (*p* < 0.05). For every increase of 10 years in age, cognitive scores decreased by 0.381 in the group with the lowest intake of dietary choline, and by 0.256 in the higher intake group. The *p*-value of the trend test was 0.0121.

## 4. Discussion

This prospective cohort study assessed the effects of dietary choline intake on cognitive function and cognitive function decline during ageing among a large-scale, community-based middle-aged and elderly Chinese sample. The average dietary choline intake fluctuated between 141.5 and 166.2 mg/d between 1997 and 2018, and was 161.1 mg/d for the baseline population of the cohort. The higher the intake of dietary choline, the better the cognitive function of middle-aged and elderly Chinese males and females, whereas dietary choline intake only slowed down the rate of cognitive decline in females during the aging process.

Due to the fact that the China Food Composition only features the choline content of some foods, there has been a lack of reports on the choline intake of the Chinese population. This study reports for the first time the dietary choline intake in the population of people aged 55 to 79 in China. Compared with other countries, the Chinese population has a lower intake of choline. In 2009–2012, the National Health and Nutrition Examination Survey (NHANES), a nationally representative cross-sectional survey of non-institutionalized civilian U.S. residents, reported that the estimated mean intake of choline from food and dietary supplements for all individuals aged ≥2 years was 317 ± 1.8 mg/d [25]. Vennemann et al. estimated the choline intake for different age classes in males and females at the European level [26]. The results showed that in Finland, France, Ireland, Italy, the Netherlands, Sweden, and the UK the average intake estimates ranged from 357 to 468 mg/d in adult males, and from 358 to 450 mg/d in elderly males, while the average intake estimates ranged from 291 to 374 mg/d in adult females, and from 284 to 377 mg/d in elderly females [26]. Compared to those countries, the dietary choline intake of the middle-aged and elderly population in China is only about half.

Although the epidemiological results of effects of choline intake on cognitive function are mixed [11,12,13,14,27,28,29], our results show higher dietary choline intake is associated with better cognitive function, which is in line with those of the study by Poly et al. [14] and Sanchez et al. [28]. The underlying mechanism of choline benefiting cognition is summarized as follows. Firstly, choline is a precursor of neurotransmitter acetylcholine (Ach) [2,3,30]. Ach receptors orchestrate the immune response in the central nervous system, and their dysregulation plays a part in the pathogenesis of Alzheimer’s disease [2]. Secondly, choline is a precursor of phosphatidylcholine (PC), which is a major constituent of all biological membranes, including neuronal and glial cell membranes [2,10]. Thirdly, choline is oxidized to betaine in two steps by choline dehydrogenase and aldehyde dehydrogenase in mitochondria. Betaine plays an important role in converting homocysteine to methionine and subsequently to S-adenosyl-methionine (SAM), which is the major methyl donor [5]. DNA methylation is highly dynamic in the adult brain and may modulate the expression of genes involved in the regulation of synaptic plasticity, learning, and memory [10].

The neuroprotection of dietary choline is widely accepted, nevertheless, emerging evidence has suggested that excess dietary choline impairs cognitive function [31]. Under the action of gut bacteria, choline is metabolized into trimethylamine (TMA), and subsequently oxidized to TMA-N-oxide (TMAO). TMAO is an acknowledged pathogenic factor of cardiovascular diseases. Recent studies have confirmed that TMAO can induce hippocampal-dependent learning and memory ability impairment with synaptic plasticity deficits by activating the mTOR signaling pathway [31]. The dietary choline intake of participants in our study was approximately 161.1 mg/d, which is far from AI. Therefore, the neuroprotection effect is greater than the cognitive impairment.

It remains unclear why the rate of cognitive decline decreased in the higher intake group of dietary choline, but not in the highest intake group in the current study. It is important to note that the reported dietary choline intake in this study was significantly lower than AI. Therefore, the neuroprotective effects of choline found in our study may not fully reflect the potential role of choline regarding cognitive function.

Our study found that the neuroprotective effects exist in females but not in males. It reports that the prevalence of AD is higher in females, representing two-thirds of cases. Mechanistic evidence points toward estrogen’s neuroprotective effects being strongly dependent on its interactions with the cholinergic system [32]. However, the “critical window hypothesis” assumes that estrogen’s putative protective effects may be restricted to early post-menopausal females only. The female subjects in our research were women aged 55 to 79 who had already gone through the menopause. The relevant studies did not conduct a stratified analysis on gender, except for Ylilauri et al. who found that total choline intake had no association with the risk of incident dementia and AD in dementia-free men aged 42~60 years from eastern Finland [32]. More evidence is needed to elucidate the gender differences in the neuroprotective effects of choline.

## 5. Conclusions

In conclusion, the findings from this large community-based prospective study suggest that dietary choline is beneficial for cognition in middle-aged and elderly Chinese, and can postpone cognition decline in middle-aged and elderly females.

## Figures and Tables

**Table 1:** Participants’ characteristics in each wave separately and the baseline.

Characteristics	Year						*p* Value	Baseline of the Cohort
	1997	2000	2004	2006	2015	2018		
Sample size, no	586	880	1507	1672	1156	895	-	1836
Age, mean ± SD (years)	61.5 ± 4.5	62.8 ± 5.1	64.0 ± 6.0	64.6 ± 6.3	70.8 ± 4.3	72.6 ± 3.5	<0.0001	60.1 ± 4.4
Sex, no (%)							0.2511	
Male	316 (53.9)	462 (52.5)	775 (51.4)	843 (50.4)	567 (49.0)	436 (48.7)		956 (50.7)
Female	270 (46.1)	418 (47.5)	732 (48.6)	829 (49.6)	589 (51.0)	459 (51.3)		931 (49.3)
Education, no (%)							<0.0001	
<middle school	442 (76.5)	605 (72.7)	1065 (70.9)	1139 (68.3)	747 (64.7)	535 (59.8)		1318 (71.7)
Middle school	64 (11.1)	111 (13.3)	241 (16.0)	281 (16.9)	247 (21.4)	219 (24.5)		279 (15.2)
>middle school	72 (12.5)	116 (13.9)	196 (13.0)	247 (14.8)	161 (13.9)	140 (15.7)		242 (13.2)
Household income, median (P_25_, P_75_) (yuan)	2315.3 (1304.7, 3773.1)	3268.6 (1693.2, 5570.3)	3748.2 (1956.7, 6894.7)	3992.4 (1953.9, 7874.5)	11,622.6 (4322.6, 23,289.0)	12,526.5 (4186.2, 26,738.0)	<0.0001	3134.2 (1607.4, 5409.5)
Energy intake, mean ± SD (kcal/d)	2358.0 ± 720.9	2244.6 ± 675.4	2204.5 ± 704.1	2228.7 ± 692.7	1883.1 ± 675.3	1927.0 ± 665.5	<0.0001	2314.2 ± 688.8
Currently smoke, no (%)							<0.0001	
No	376 (65.6)	587 (67.2)	1045 (69.7)	1215 (72.8)	900 (78.0)	722 (81.1)		1262 (67.5)
Yes	197 (34.4)	286 (32.8)	455 (30.3)	455 (27.2)	254 (22.0)	168 (18.9)		608 (32.5)
Alcohol intake, no (%)							<0.0001	
No	379 (65.2)	599 (69.6)	1071 (71.5)	1189 (71.5)	905 (78.8)	718 (80.9)		1259 (67.6)
Yes	202 (34.8)	262 (30.4)	427 (28.5)	474 (28.5)	243 (21.2)	169 (19.1)		603 (32.4)
Dietary choline intake, median (P_25_, P_75_) (mg/d)	155.5 (82.2, 228.9)	160.5 (94.2, 253.7)	154.3 (96.3, 244.0)	166.2 (103.8, 253.8)	141.5 (82.6, 212.6)	142.9 (87.8, 219.0)	<0.0001	161.1 (91.9, 243.6)

## Data Availability

The CHNS datasets used during the current study are partly available at http://www.cpc.unc.edu/projects/china/data, accessed on 30 July 2024.
